# Dose–Response Relationship between Western Diet and Being Overweight among Teachers in Malaysia

**DOI:** 10.3390/nu12103092

**Published:** 2020-10-11

**Authors:** Jui Yee Eng, Foong Ming Moy, Awang Bulgiba, Sanjay Rampal

**Affiliations:** Centre for Epidemiology and Evidence-based Practice, Department of Social and Preventive Medicine, Faculty of Medicine, University of Malaya, Kuala Lumpur 50603, Malaysia; joey.eng.jy@gmail.com (J.Y.E.); awang@ummc.edu.my (A.B.); srampal@ummc.edu.my (S.R.)

**Keywords:** dietary pattern, overweight, obesity, Western diet, Prudent diet, dose–response

## Abstract

The rising prevalence of overweight and obesity is partly due to nutrition transition. The reported association between dietary patterns and overweight/obesity has been controversial because of inconsistent results and weak observed associations. Although it has been hypothesized that an unhealthy diet can increase obesity risk, none of the previous studies have examined the dose–response association using nonlinear dose–response analyses. This study aimed to examine the dose–response association between major dietary patterns and overweight/obesity. This was a cross-sectional study involving teachers selected through stratified multistage sampling from public schools in three Malaysian states. Dietary intake was assessed using a food frequency questionnaire, and two major dietary patterns (Western and Prudent diet) were extracted using factor analysis. Logistic regression followed by trend analysis was used to test the difference in odds of overweight and obesity in each quintile of diet score. A further analysis using restricted cubic spline models was performed to examine the dose–response associations of dietary patterns with odds of overweight/obesity. The logistic regression analysis showed that participants with the highest quintile of Western diet score were 1.4 times more likely to be overweight/obese compared to those in the lowest quintile (95% CI: 1.11, 1.83, *p*-trend < 0.001). The odds of overweight/obesity showed a significant increasing trend across quintiles of Western diet among both men and women (*p*-trend < 0.001). In the dose–response analysis, a positive linear association (P_nonlinearity_ = 0.6139) was observed where overweight/obesity was more likely to occur among participants with a Western diet score greater than a mean score of zero. There was an inverse trend of odds of overweight/obesity across quintiles of Prudent diet score, significant only for men (*p* for trend < 0.001). Linear association was found between Prudent diet score and odds of overweight/obesity among both men (P_nonlinearity_ = 0.6685) and women (P_nonlinearity_ = 0.3684) in the dose–response analysis. No threshold at the level of adherence to Prudent diet was linked to odds of overweight/obesity. Dose–response analysis indicated that women with a Western diet score greater than zero were more likely to be overweight or obese among women. In men, higher adherence to Western diet was associated with increased odds of overweight/obesity, while greater adherence to Prudent diet decreased the odds of overweight/obesity. Promoting and enhancing the consumption of Prudent diet and limit in Western diet may be used to guide the development of evidence-based diet interventions to curb overweight and obesity.

## 1. Introduction

Obesity is one of the leading global public health problems worldwide. According to the World Health Organization, the worldwide prevalence of obesity has almost tripled between 1975 and 2016. In 2016, 13% (650 million) of the world’s adult population was obese [1]. In Malaysia, data from the National Health and Morbidity Survey (NHMS) estimated that the number of adults aged 18 and above with overweight and obesity (BMI ≥ 25 kg/m^2^) has increased from 44.5% in 2011 to 50.1% in 2019.

Over the past two decades, Malaysia has undergone a rapid nutrition transition characterized by a shift away from the traditional grain-based diet (high in complex carbohydrates and fiber) to a Westernized diet, which is high in animal-based foods, high-fat foods, sugar-sweetened beverages, and lower in fiber [2,3]. Goh and colleagues suggest that [3] the nutrition transition has contributed to the development of obesity and diet-related conditions such as diabetes mellitus, hypertension, and hyperlipidemia. Many investigators have determined the relationships of nutrients or food groups with the prevalence of obesity [4,5,6,7,8]. However, these studies may limit the impact of diet on obesity due to the complexity of the human diet and the potential synergistic effect of individual nutrients or food groups [9]. Dietary pattern analysis has been proposed as a better approach in assessing diet quality than a single nutrient or food group. Many studies have been conducted to investigate the association between dietary patterns and obesity [10,11,12,13,14]. A meta-analysis of 18 studies conducted in both Western and Asian populations reported that higher adherence to a Prudent diet was associated with a 36% (OR = 0.64; 95% CI: 0.52, 0.78) lower odds of being overweight or obese [15].

By contrast, those with the highest adherence to a Western diet had 65% (OR = 1.65; 95% CI: 1.45, 1.87) higher odds of being overweight or obese compared to those with the lowest adherence [15]. Other specific dietary patterns such as Mediterranean diet were found to be associated with a lower risk of obesity [16,17,18], while the Traditional diet showed an inconsistent association [19,20,21,22]. However, these findings may not apply to Malaysians as culturally specific dietary patterns are likely to play a crucial role. In addition, the quality of evidence on the associations between dietary pattern and risk of obesity needs to be taken into account, and the optimal intakes associated with the greatest risk increase or reduction need to be clarified.

Previous studies reported the association between dietary patterns and obesity. Still, these studies were mostly limited to dividing the dietary pattern score into percentiles, which could not show the dose–response association. Restricted cubic spline combines a spline function with a generalized linear model such as logistic regression to visually present the threshold of diet score that is associated with increased or decreased odds of overweight/obesity. The findings are important to support current public health dietary guidelines for the dietary prevention of obesity. However, the dose–response association between dietary patterns and overweight/obesity among Malaysians has not been studied. Therefore, this study aimed to examine the association between dietary patterns and overweight/obesity and to explore their dose–response association using a restricted cubic spline (RCS) model.

## 2. Materials and Methods

### 2.1. Study Design

This cross-sectional study is a substudy under Clustering of Lifestyle Risk Factors and Understanding its Association with Stress on Health and Wellbeing of School Teachers (CLUSTer) study. CLUSTer is a cohort study with a primary focus on the interaction between work-related stress and clustering of lifestyle risk factors on health and wellbeing among schoolteachers in Peninsular Malaysia. Details about the study protocol have been published elsewhere [23].

The sample size was calculated using the Open Epi version 2.3.1 software [24]. Mu and colleagues reported that higher adherence to a Western diet was 1.65 (95% CI: 1.45, 1.87) times more likely to result in being overweight or obese compared with those with low adherence [15]. With a confidence level of 95%, power of 80%, percentage of unexposed with an outcome of 36%, and prevalence of overweight and obesity among Malaysian adults of 47.7%, the minimum sample size required was 1375. The final sample size determined to accommodate a nonresponse rate of 30% was 1788 participants. The participants were selected through a multistage sampling procedure from all districts of six randomly selected states in Peninsular Malaysia. About 70% of public schools were randomly selected, and all teachers were invited to participate. However, only data collected between September 2014 to November 2015 was used in the current study. This is because dietary data were not available at the time when the initial data collection was conducted (from January 2013 through August 2014). Ethical approval was obtained (Reference Number: 950.1) from the Medical Ethics Committee of the University Malaya Medical Centre (UMMC). Approval was also granted by the Ministry of Education, the relevant State Education Department, and principals of all invited schools. Written informed consent was obtained from all participants.

### 2.2. Sociodemographic and Lifestyle Characteristics Questionnaire

Sociodemographic and lifestyle characteristics were obtained using a self-administered questionnaire. Basic background information about the participants’ age, sex, ethnicity, education level was assessed. Information on physical activity was collected using a Malay version of International Physical Activity Questionnaire-Short Form [25,26]. Smoking status among participants was assessed, and they were classified as current smokers, former smokers, and having never smoked. The completeness of returned questionnaires was checked by the research assistant upon collection.

### 2.3. Dietary Pattern Assessment

Dietary intakes for the past one year were collected using a 136-item food frequency questionnaire (FFQ) that has been developed and validated for the study population (data not published). The FFQ was in both Malay and English; it took approximately 30–45 min to complete. All questionnaires were personally distributed to participants, and verbal and detailed written instructions on how to fill out the FFQ were given. Participants were asked to specify the number of times per day, week, month or year. The participants were also asked to determine the amount they typically consumed for each food item. Color photographs of serving size for selected food items were attached to improve the ability of serving size estimation. All returned questionnaires were visually inspected before entered by scanning using the Teleform program.

Dietary data were assessed among 6742 participants in the CLUSTer Study, but only 4618 participants with completed data were included in the dietary pattern analysis. Dietary patterns were identified using factor analysis, based on the intake of the 43 food groups, using principal component analysis as an extraction method followed by varimax rotation. Two major dietary patterns were identified: Western and Prudent diet. Diet scores were calculated using the Bartlett method [27], and each participant received a score for each dietary pattern. A positive score indicated higher adherence, and a negative score indicated lower adherence to that dietary pattern. The detailed description of the identification of dietary patterns has been published [28].

### 2.4. Anthropometric Measurements

Height was measured using a SECA stadiometer (SECA 217, Hamburg, Germany) accurate to 0.1 cm without shoes and both feet flat on the floor and heels, buttock, shoulder blades, as well as the back of the head touching the measurement surface. Body weight accurate to 0.1 kg was assessed using a Bioelectrical Impedance Meter (Tanita, TBF-300A Body Composition Analyzer, Tanita Corporation, Tokyo, Japan). The participants were measured in light clothing and with bare feet. All the anthropometry data were manually entered, and any erroneous data were corrected and documented. Body mass index (BMI) was calculated as weight in kilograms divided by height in meters and dichotomized as normal/underweight (BMI < 25.0 kg/m^2^) and overweight/obese (BMI ≥ 25 kg/m^2^) based on WHO criteria [29].

### 2.5. Missing Data

All the variables of interest were identified, and the proportions of missing data for all variables of interest were assessed. Further examination indicated that the data were missing at random. Therefore, missing data were imputed using multiple imputation by chained equation [30,31]. With approximately 54% of overall missing data, 50 imputations [31] with burn-in of 1000 iterations were generated. The imputation model was built using the following variables: age, ethnicity, urban status, education level, smoking status, physical activity, weight, height, waist circumference, hip circumference, fasting blood glucose, blood pressure, lipid profile, self-reported diabetes, self-reported medication for diabetes, dietary pattern scores, and total energy intake. The imputation was performed separately by sex so that interaction effects on gender could be included in the analysis model [31]. The imputed data were used in all analyses.

### 2.6. Statistical Analysis

The participants were classified into five groups according to the quintiles of the dietary pattern scores. To assess the trend associations in the characteristics of participants among the quintiles of the Western and Prudent diets, linear regression analysis for continuous variables, and the Mantel–Haenszel chi-square test for categorical variables were used, assigning ordinal numbers 1 to 5 to represent the lowest to highest quintiles of the diet scores.

Separate analysis models were performed to assess the associations of BMI with each dietary pattern for men and women. The logistic regression model was used to estimate the odds of overweight/obesity with dietary patterns as a quintile score (with the lowest quintile as a reference). The median value of each quintile was used to test for linear trends across quintiles in both linear and logistic regression models. All analyses were adjusted according to two models. The first model was adjusted for age, sex, and daily energy intake. The second model was further adjusted for ethnicity, education, urban status, smoking status, physical activity level, and daily energy intake. Further analyses using RCS with 5 knots were performed to explore the dose–response associations between both dietary patterns and odds of overweight/obesity. The *p*-value for nonlinearity was determined by testing the null hypothesis that the coefficient of the second spline is equal to zero. All data analyses were conducted using Stata version 13.0 [32], and the significance level was set at 0.05.

## 3. Results

The CLUSTer study was conducted between September 2014 and November 2015, and a total of 7138 participants consented. However, 396 participants withdrew from the study due to insufficient time to complete the questionnaires. There was a total of 6742 teachers included in the analyses. Two major dietary patterns were identified: Western and Prudent [23]. Western diet explained 15.2% of the variance and was characterized by a high intake of refined carbohydrates, animal-based food, sugar-sweetened food, and beverages, as well as fast food. Prudent diet which explained 10.9% of the variance and had a high loading for pulses, legumes, vegetables, and fruits.

The overall mean age of the participants was 40.3 years (standard error = 0.1) (Table 1). There were more women than men (82.6% versus 17.4%). Most of the participants were Malays (67.4%), followed by Chinese (22.4%), Indians (9.6%), and others (0.7%). About 67.1% of them were teaching in an urban area. About 93.4% of the participants had never smoked, while the percentages of former and current smokers were 4.3% and 2.3%, respectively. A quarter (24.8%) of the participants had a low physical activity level, about 54.9% of them were moderately active, and 20.3% reported a high physical activity level. More than half of the participants were overweight or obese (52.1%), and about 46.2% had abdominal obesity. Significant trends across quintiles of the Western diet were observed for age, sex, ethnicity, urban status, physical activity, smoking status, daily energy intake, BMI, and waist circumference. The results were similar for the Prudent diet. However, physical activity status and BMI did not significantly differ across quintiles of the Prudent diet.

Table 2 presents the results of logistic regression and trend test for overweight/obesity with each dietary pattern. In the adjusted logistic regression model, the highest quintile of the Western diet (vs. lowest) had 1.4 times greater odds of overweight/obesity (95% CI = 1.11, 1.83) among all participants. A statistically significant trend toward increasing odds existed across quintiles for the Western diet (*p*-trend < 0.001). Results were generally similar when stratified by sex, with similar associations between Western diet and overweight/obesity in both men and women. For the Prudent diet, the estimated odds ratios for overweight/obesity were greater than one across all quintiles among all participants, but only the second quintile was significant (OR: 1.24; 95% CI: 1.02, 1.50). There was a significant decreasing trend for odds of overweight/obesity across quintiles of the Prudent diet (*p*-trend < 0.001). Although no significant association was observed between Prudent diet and overweight/obesity among men, the trend analysis suggested that higher adherence to the Prudent diet was associated with lower odds of overweight/obesity (*p*-trend < 0.001). The odds ratios and test for trend (*p*-trend = 0.735) across quintiles of the Prudent diet were not significant among women.

**Figure 1:**
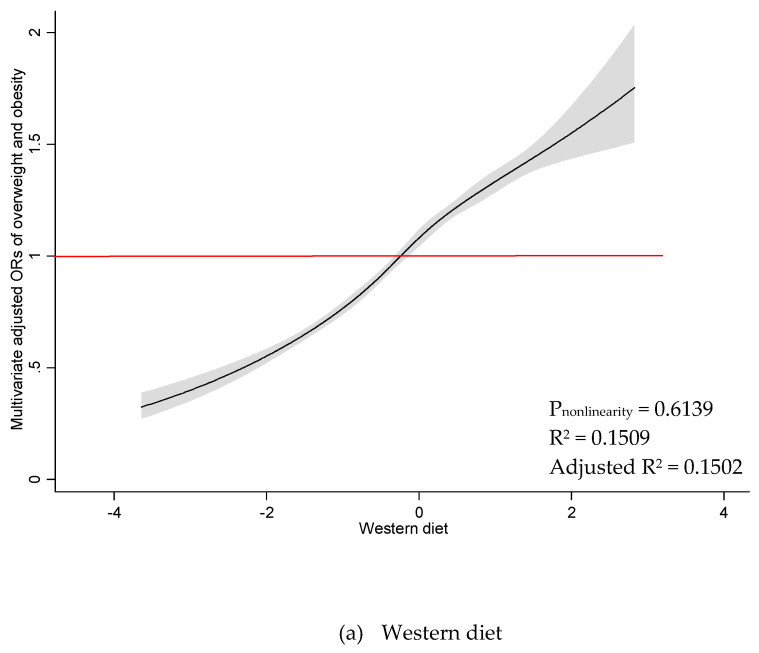

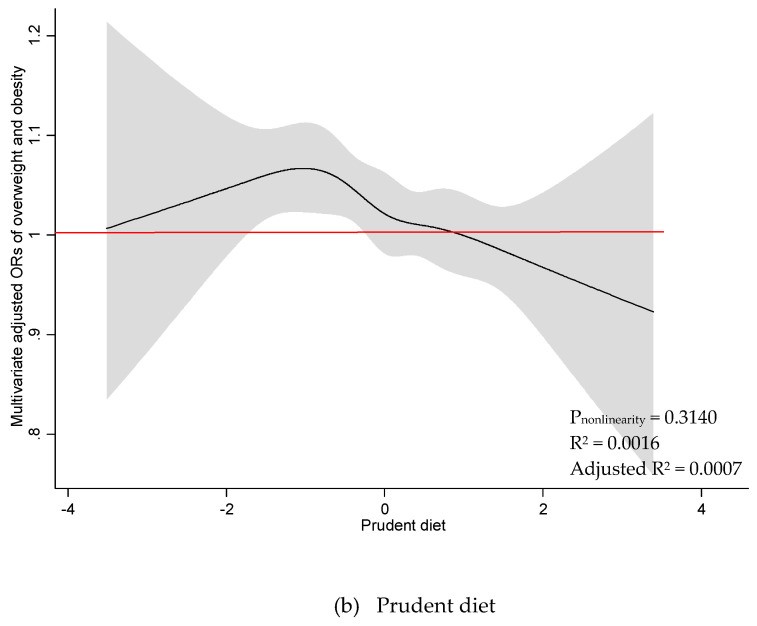
Restricted cubic spline plots between dietary patterns and multivariate adjusted odds ratio of overweight and obesity among all participants. (**a**) Western diet and (**b**) Prudent diet. Results obtained by multivariable logistic regression with restricted cubic splines with five knots. Solid black lines indicate odds ratio, and shaded areas indicate 95% CI.

**Figure 2:**
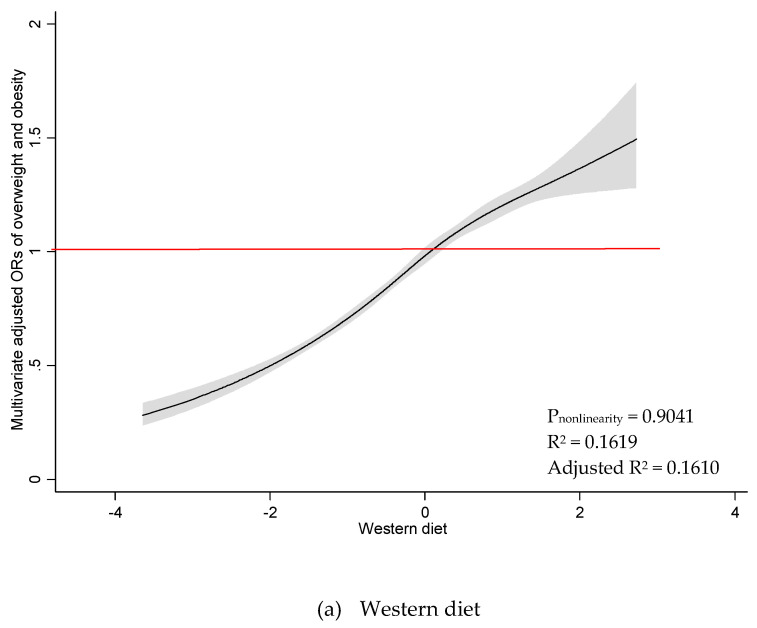

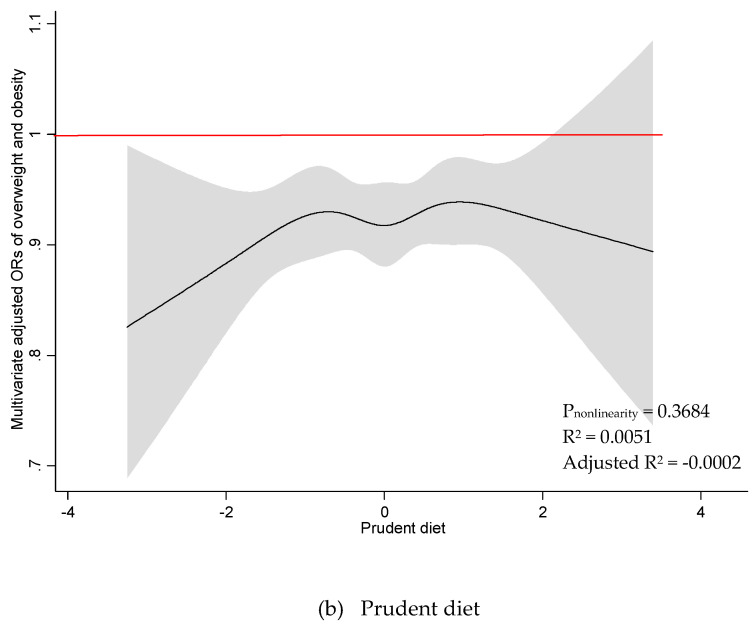
Restricted cubic spline plots between dietary patterns and multivariate adjusted odds ratio of overweight and obesity among women. (**a**) Western diet and (**b**) Prudent diet. Results obtained by multivariable logistic regression with restricted cubic splines with five knots. Solid black lines indicate odds ratio, and shaded areas indicate 95% CI.

**Figure 3:**
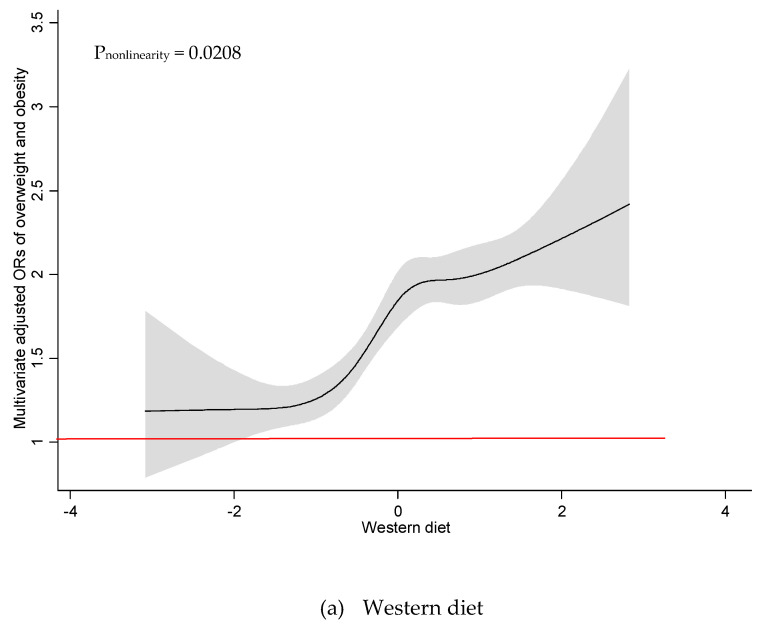

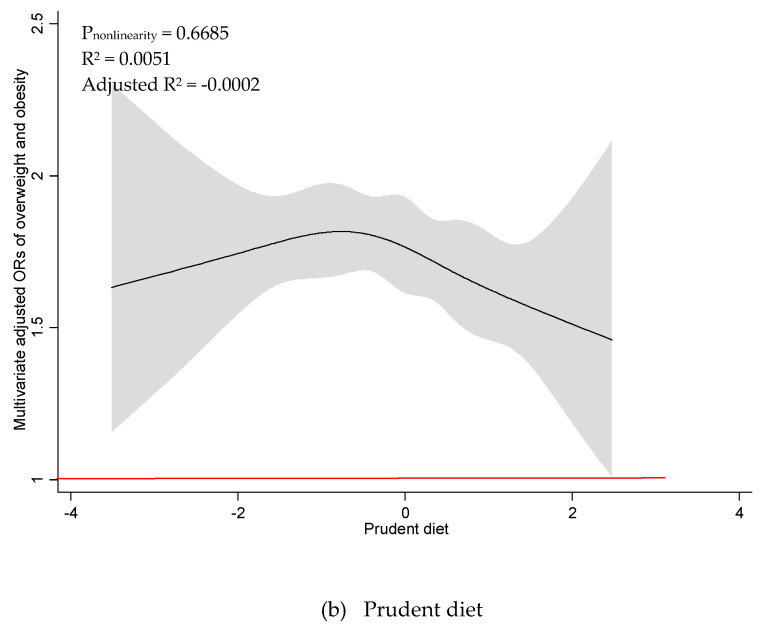
Restricted cubic spline plots between dietary patterns and multivariate adjusted odds ratio of overweight and obesity among all men. (**a**) Western diet and (**b**) Prudent diet. Results obtained by multivariable logistic regression with restricted cubic splines with five knots. Solid black lines indicate odds ratio, and shaded areas indicate 95% CI.

As illustrated in Figure 1, there was a significant linear association between Western diet and odds of overweight/obesity among all participants (P_nonlinearity_ = 0.6139). Odds of overweight/obesity were found to increase with higher adherence to the Western diet, where a greater likelihood of overweight/obesity was reported when the Western diet scored above mean of zero. A similar association was observed among women (Figure 2) (P_nonlinearity_ = 0.9041). There was a nonlinear association between Western diet and odds of overweight/obesity among men (P_nonlinearity_ = 0.0208), and no significant dose threshold was observed (Figure 3). Linear associations between the Prudent diet and odds of overweight/obesity were observed among all participants (P_nonlinearity_ = 0.3140), as well as for men (P_nonlinearity_ = 0.6685) and women (P_nonlinearity_ = 0.3684) separately. The results suggest that the Prudent diet contributed to odds of overweight/obesity linearly among both men and women. The RCS model provided better a fit compared to the linear model for the Western diet. In contrast, linear models indicated a better fit than RCS models for the Prudent diet.

## 4. Discussion

Although the causes of obesity are likely to be multifactorial, diet plays an important role. Western diet could be an important contributory factor. Both trend and dose–response analyses showed linear association between the Western diet and odds of overweight/obesity among women. Higher odds of overweight/obesity were reported among women who scored a Western diet score above a mean of zero. This could be explained by the fact that those with a Western diet score greater than zero had shifted their energy intake from carbohydrates toward protein (data not shown). Animal-based proteins such as red meat and processed meat were reported to be associated with the development of obesity [33,34,35]. Higher intake of animal protein may have a major impact on the increased odds of overweight/obesity in the Western diet. Although the trend and dose–response analyses yielded contradictory linearity results, both analyses showed positive directions. A weak linear correlation was observed in the linear analysis, which may indicate a nonlinear association. No threshold at which a higher Western diet score was linked to odds of overweight/obesity among men was observed. Generally, higher adherence to the Western diet was associated with higher energy intake, which explained weight gain and increased risks of overweight and obesity.

In the trend analyses, a significant positive trend was observed for all participants and men. These findings were in agreement with the nonlinear model. There was a discrepancy between the linear and nonlinear model between the Prudent diet and odds of overweight/obesity for women. Previous studies have reported that high vegetable and fruit intake was inversely associated with body weight [36,37,38]. However, no association between vegetable intake and risk of overweight or obese was observed in other studies [34,39,40]. Higher adherence to the Prudent diet in the current study may not have a significant impact on overweight/obesity even though it was loaded with pulses, legumes, green leafy vegetables, cruciferous vegetables, other vegetables, root vegetables, starchy vegetables, mixed vegetables, vegetable soups, and fruits. The observed Prudent diet was high in total calorie, which may lead to overweight/obesity. An examination of the nutrient composition revealed an increase in total calories consumed with higher adherence to the Prudent diet, with total fat being the dominant source of calories (data not shown). This could be explained by the heterogeneity of our local food culture, where the use of palm oil and coconut milk in cooking is common, which contributed to an increase in overall calories from fat in the diet. In addition, women may be more concerned with diet quality, having high adherence in the Prudent diet, but not in the total calorie of the diet. Although linear associations were reported in the linear models for all participants and men, weak adjusted R-squared values indicated the Prudent diet has a low variance effect on the odds of overweight/obesity. A weak adjusted R-square value indicated the Prudent diet has a low variance effect on the odds of overweight/obesity for all participants as well as among women. In summary, the effect of a Prudent diet on overweight and obesity status maybe less apparent in the current study.

Previous studies have generally characterized the Western diet as having high intakes of red meat, processed meat, refined grains, sugar-sweetened food and beverages, high-fat dairy products, and deep-fried foods [41,42,43]. The characteristics of the Western diet identified in the current study were similar to those of the Western diet observed in previous studies. The Prudent diet identified in previous studies was generally high in whole grains, vegetables, fruits, legumes, pulses, low-fat dairy, poultry, and fish. Nevertheless, the Prudent diet derived in the current study seems to reflect only part of the general Prudent diet, such as higher intakes of vegetables, fruits, legumes, and pulses. Therefore, careful attention is needed when interpreting the findings and not to assume that a Prudent diet identified in the current study is similar to others.

Although the nonlinear association between dietary patterns in relation to obesity has not previously been studied, a recent meta-analysis reported on the dose–response association of selected food groups with the risk of overweight and obesity. An increased risk was observed for refined grains and sugar-sweetened beverages [34]. These food items were found to be highly loaded on the Western diet in the current study. This shows that refined grains and sugar-sweetened beverages probably have a great impact on the development of obesity. A similar meta-analysis also suggests that a high intake of whole grains, vegetables, and fruit reduced the risk for overweight and obesity. In theory, the combined effect of these components may be beneficial in preventing obesity. The weak association between Prudent diet and odds of overweight and obesity in the current study may be explained by the local dietary habits where whole grains were not commonly consumed among the Malaysian population [44,45]. Additionally, increased consumption of palm oil in the diet may contribute to the high-calorie intake among Malaysians [46]. Such dietary practices coupled with physical inactivity may contribute to high prevalence of overweight and obesity.

Several limitations need to be addressed while interpreting the results. Causality between dietary patterns and obesity cannot be established due to the study’s cross-sectional design. All participants had tertiary education, with more Malays and women in the sample than found in the demographic composition of Malaysia. Hence, this may limit the extrapolation of results to the general population in Malaysia. In addition, the diet data were collected in 2014–2015, and there may have been some changes in eating habits over the past 5 years. The recall method for FFQ may over- or underestimate dietary intake. To reduce potential recall bias, we offered food pictures to participants to recall the portion size of the food consumed. Future studies with a prospective cohort design and a heterogeneous sample with a good mix of sex, ethnic, and socioeconomic status are recommended.

Notwithstanding its limitations, this study has several strengths. The study has one of the largest samples with dietary pattern exposures in Malaysia, which has not been assessed in previous local studies. Furthermore, analyses were performed using multiple imputed data to avoid bias estimates attributable to missing data. A comprehensive questionnaire was used to control for important confounders such as anthropometric, sociodemographic, and lifestyle factors. To the best of our knowledge, no other studies have explored the association between empirically dietary patterns and odds of overweight and obesity using RCS models to provide a better assessment of the potential dose–response association. Here, we have included comprehensive analyses including both linear and nonlinear dose–response analyses. Dose–response analysis is better to reflect the overall trend for the odds of overweight and obesity because the continuous odds ratio is more accurate than the linear model. Additionally, dose–response analysis enables us to predict a threshold score, which will contribute to the higher odds of overweight and obesity. The current study has added evidence to the existing literature with a paucity of dose–response association between dietary patterns and obesity.

## 5. Conclusions

In summary, our findings suggest that women who reported a Western diet score above mean of zero were more likely to become overweight or obese. Although no dose–response association was obtained among men, the results suggest a higher adherence to a Western diet was associated with increased odds of overweight and obesity. The effects of a Prudent diet on overweight and obesity status were less apparent in the current study. Shifting dietary patterns along with the scales of a particular dietary pattern by making small and relevant suggestions for change is more achievable and sustainable rather than considerable changes to a less familiar dietary pattern. Promotion of diet change based on tailored interventions that match individual dietray pattern has been shown to be effective for encouraging weight loss. Innovative interventions can be easily replicated and sustained linking primary care practices with home-based programs.

## Figures and Tables

**Table 1:** General characteristics of the study participants across quintiles of dietary pattern scores.

Characteristics	Overall	Western Diet				Prudent Diet			
		Q1	Q3	Q5	*p*-Trend ^a^	Q1	Q3	Q5	*p*-Trend ^a^
Age (years)	40.3 (9.0)	42.5 ± 0.3	40.3 ± 0.3	38.2 ± 0.3	<0.001	38.9 ± 0.3	40.6 ± 0.3	40.9 ± 0.3	<0.001
Gender									
Men	1174 (17.4)	16.2 (1.3)	17.9 (1.3)	25.4 (1.5)	<0.001	24.8 (1.5)	22.4 (1.4)	12.0 (1.2)	<0.001
Women	5568 (82.6)	21.2 (0.6)	20.2 (0.6)	18.5 (0.5)		19.0 (0.6)	19.3 (0.5)	22.0 (0.6)	
Race									
Malay	4542 (67.4)	11.9 (0.5)	20.2 (0.7)	26.9 (0.7)	<0.001	22.5 (0.7)	21.3 (0.7)	14.1 (0.6)	<0.001
Chinese	1507 (22.4)	37.3 (1.4)	18.9 (1.1)	5.5 (0.7)		14.6 (1.0)	16.9 (1.0)	32.3 (1.4)	
Indian	649 (9.6)	38.6 (2.4)	18.9 (1.9)	3.0 (0.8)		15.7 (1.8)	15.9 (1.8)	34.4 (2.3)	
Others	44 (0.7)	44.2 (8.6)	20.5 (6.9)	7.2 (4.1)		9.5 (5.7)	21.9 (7.0)	34.5 (8.7)	
Education level									
Diploma	1517 (22.5)	35.5 (1.5)	18.2 (1.2)	8.7 (0.8)	<0.001	16.9 (1.2)	17.2 (1.1)	29.6 (1.4)	<0.001
Degree	4859 (72.1)	15.6 (0.6)	20.1 (0.6)	23.2 (0.6)		20.9 (0.6)	21.0 (0.6)	17.0 (0.6)	
Master/PhD	366 (5.4)	20.9 (2.3)	22.4 (2.3)	18.5 (2.3)		20.2 (2.3)	15.9 (2.1)	23.5 (2.4)	
Urban status									
Urban	4521 (67.1)	15.6 (0.9)	20.0 (0.9)	24.2 (1.0)	<0.001	21.9 (1.0)	20.3 (1.0)	16.4 (0.9)	<0.001
Rural	2221 (32.9)	22.7 (0.7)	19.7 (0.6)	17.5 (0.6)		19.1 (0.6)	19.6 (0.6)	22.1 (0.7)	
Physical activity level									
Low	1252 (24.8)	24.4 (1.3)	19.8 (1.2)	17.1 (1.2)	<0.001	22.0 (1.3)	19.5 (1.3)	19.6 (1.2)	0.263
Moderate	2770 (54.9)	19.8 (0.8)	20.4 (0.8)	19.7 (0.8)		18.3 (0.8)	19.1 (0.8)	22.3 (0.9)	
High	1025 (20.3)	17.9 (1.3)	19.9 (1.4)	20.7 (1.4)		20.3 (1.4)	22.5 (1.5)	17.0 (1.3)	
Smoking status									
Never smoked	5249 (93.4)	20.9 (0.5)	19.9 (0.5)	18.9 (0.5)	<0.001	19.6 (0.5)	19.5 (0.5)	21.1 (0.5)	<0.001
Former smoker	129 (2.3)	13.6 (3.1)	14.5 (3.5)	35.6 (4.6)		25.3 (4.3)	25.8 (3.9)	8.4 (2.7)	
Current smoker	241 (4.3)	12.7 (2.2)	20.5 (2.6)	27.3 (3.0)		24.7 (3)	24.3 (2.8)	7.7 (1.9)	
Daily energy (kcal/day)	2874.1 ± 21.7	1519.9 ± 17.7	2923.6 ± 38.9	3873.3 ± 46.3	<0.001	1804.4 ± 24.2	3373.8 ± 53.0	2971.6 ± 43.9	<0.001
Carbohydrate (%TE) ^b^	48.3 ± 0.3	49.3 ± 0.3	48.4 ± 0.2	47.1 ± 0.2	<0.001	51.8 ± 0.2	48.4 ± 0.2	44.3 ± 0.2	<0.001
Protein (%TE) ^b^	18.2 ± 0.1	16.7 ± 0.1	18.4 ± 0.1	19.2 ± 0.1	<0.001	17.7 ± 0.1	18.3 ± 0.1	18.7 ± 0.1	<0.001
Total fat (%TE) ^b^	33.4 ± 0.1	33.9 ± 0.2	33.1 ± 0.2	33.6 ± 0.1	< 0.106	30.4 ± 0.2	33.3 ± 0.2	36.8 ± 0.2	<0.001
BMI (kg/m^2^)	25.2 (22.2,28.7)	25.1 ± 0.1	25.8 ± 0.2	26.6 ± 0.2	<0.001	25.7 ± 0.2	26.0 ± 0.2	25.6 ± 0.2	0.451
BMI classification ^c^	276 (4.1)								
Underweight/normal	2944 (43.8)	23.4 (0.8)	19.8 (0.8)	17.0 (0.7)	<0.001	20.7 (0.8)	19.3 (0.7)	21.5 (0.8)	0.506
Overweight	2253 (33.5)	18.5 (0.9)	20.3 (1.0)	20.8 (0.9)		19.8 (1.0)	20.3 (1.0)	19.1 (0.9)	
Obese	1248 (18.6)	15.9 (1.2)	18.7 (1.2)	24.4 (1.3)		18.5 (1.3)	20.5 (1.3)	19.1 (1.2)	

^a^*p*-values for trend were calculated using the quintile median values. ^b^ Percentage of total energy intake. TE: total energy. ^c^ Defined according to WHO classification (BMI of less than 18.5 kg/m^2^ considered underweight, 18.5 to 24.9 normal weight, 25.0 to 29.9 overweight, and above 30 obese). Data are presented as mean ± standard error for continuous variables and row percentage, % (standard error) for categorical variables. Q1 is the lowest quintile and Q5 is the highest quintile.

**Table 2:** Odds of overweight/obesity according to adherence to the Western and Prudent diet among men and women.

	Western Diet			Prudent Diet		
	All Participants	Men	Women	All Participants	Men	Women
**Model 1 ^a^**						
**Quintiles of score ^c^**						
Quintile 1	Ref.	Ref.	Ref.	Ref.	Ref.	Ref.
Quintile 2	1.37 (1.14, 1.65)	1.24 (0.74, 2.06)	1.39 (1.13, 1.70)	1.10 (0.91, 1.33)	1.08 (0.70, 1.68)	1.12 (0.91, 1.38)
Quintile 3	1.62 (1.32, 1.99)	1.66 (0.97, 2.85)	1.61 (1.29, 2.01)	0.95 (0.78, 1.15)	1.02 (0.64, 1.64)	0.94 (0.76, 1.17)
Quintile 4	1.96 (1.56, 2.45)	1.70 (0.98, 2.94)	2.01 (1.57, 2.57)	0.92 (0.75, 1.13)	0.77 (0.47, 1.28)	0.96 (0.76, 1.20)
Quintile 5	2.30 (1.83, 2.88)	1.87 (1.07, 3.28)	2.40 (1.86, 3.10)	0.82 (0.68, 0.99)	0.71 (0.42, 1.21)	0.84 (0.68, 1.03)
*p*-trend	<0.001	<0.001	<0.001	<0.001	<0.001	0.041
R-squared	0.2231	0.0732	0.3056	0.0043	0.1208	0.0037
**Model 2 ^b^**						
**Quintiles of score ^c^**						
Quintile 1	Ref.	Ref.	Ref.	Ref.	Ref.	Ref.
Quintile 2	1.16 (0.95, 1.41)	1.15 (0.67, 1.96)	1.16 (0.93, 1.44)	1.24 (1.02, 1.50)	1.19 (0.76, 1.87)	1.25 (1.01, 1.55)
Quintile 3	1.22 (0.98, 1.51)	1.36 (0.77, 2.42)	1.19 (0.94, 1.51)	1.15 (0.94, 1.41)	1.19 (0.73, 1.93)	1.14 (0.91, 1.43)
Quintile 4	1.31 (1.03, 1.67)	1.37 (0.76, 2.50)	1.30 (1.00, 1.70)	1.20 (0.97, 1.49)	0.97 (0.56, 1.66)	1.24 (0.98, 1.58)
Quintile 5	1.43 (1.11, 1.83)	1.39 (0.75, 2.60)	1.45 (1.09, 1.91)	1.18 (0.96, 1.45)	0.93 (0.52, 1.67)	1.22 (0.97, 1.52)
*p*-trend	<0.001	<0.001	<0.001	<0.001	<0.001	0.735
R-squared	0.1077	0.0417	0.1160	0.0043	0.0043	−0.0004

Data presented as odds ratio (95% CI). ^a^ Model 1 was adjusted for age, sex, and daily energy intake. ^b^ Model 2 was further adjusted for ethnicity, education, urban status, smoking status, and physical activity level. ^c^ Quintiles of dietary pattern scores; Q1 is the lowest quintile (referent category) and Q5 is the highest quintile.
